# Evaluating the Photoprotective Effects of Ochre on Human Skin by *In Vivo* SPF Assessment: Implications for Human Evolution, Adaptation and Dispersal

**DOI:** 10.1371/journal.pone.0136090

**Published:** 2015-09-09

**Authors:** Riaan F. Rifkin, Laure Dayet, Alain Queffelec, Beverley Summers, Marlize Lategan, Francesco d’Errico

**Affiliations:** 1 Institute for Archaeology, History, Culture and Religion, University of Bergen, Bergen, Norway; 2 Evolutionary Studies Institute, University of the Witwatersrand, Johannesburg, South Africa; 3 Centre National de la Recherche Scientifique, Unité Mixte de Recherche, University of Bordeaux, Pessac, France; 4 Photobiology Laboratory, Department of Pharmacy, University of Limpopo, Medunsa, South Africa; University of Oxford, UNITED KINGDOM

## Abstract

Archaeological indicators of cognitively modern behaviour become increasingly prevalent during the African Middle Stone Age (MSA). Although the exploitation of ochre is viewed as a key feature of the emergence of modern human behaviour, the uses to which ochre and ochre-based mixtures were put remain ambiguous. Here we present the results of an experimental study exploring the efficacy of ochre as a topical photoprotective compound. This is achieved through the *in vivo* calculation of the sun protection factor (SPF) values of ochre samples obtained from Ovahimba women (Kunene Region, Northern Namibia) and the Palaeozoic Bokkeveld Group deposits of the Cape Supergroup (Western Cape Province, South Africa). We employ visible spectroscopy, energy-dispersive X-ray fluorescence (ED-XRF), X-ray diffraction (XRD) and granulometric analyses to characterise ochre samples. The capacity of ochre to inhibit the susceptibility of humans to the harmful effects of exposure to ultraviolet radiation (UVR) is confirmed and the mechanisms implicated in the efficacy of ochre as a sunscreen identified. It is posited that the habitual application of ochre may have represented a crucial innovation for MSA humans by limiting the adverse effects of ultraviolet exposure. This may have facilitated the colonisation of geographic regions largely unfavourable to the constitutive skin colour of newly arriving populations.

## Introduction

Climate exerts a significant influence on ecosystems, communities and populations [1–4]. Of the climate-driven selective pressures that operated during the evolutionary history of the genus *Homo*, negotiating the risks and benefits of persistent exposure to sunlight presented an enduring challenge. Sunlight is an essential environmental factor in nearly all ecosystems [5, 6]. Most important in terms of human health are long-wave ultraviolet A (UVA) (315–400 nm) and medium-wave UVB (280–315 nm) radiation [7]. Whereas UVA causes DNA damage, skin aging and mild erythema in large doses, UVB is responsible for sunburn with subsequent DNA damage and skin cancers. Short-wave UVC (200–280 nm) is the most dangerous type of UVR but is largely absorbed by the ozone layer [6].

The beneficial effects of moderate sunlight exposure are numerous and several positive correlations exist between adequate UVR exposure, vitamin D synthesis and human fertility [8, 9, 10]. Vitamin D (the active form being 1.25 dihydroxyvitamin D_3_ or 1.25[OH]_2_D_3_) in turn reduces the incidence of rheumatoid arthritis, coronary heart disease, diabetes, multiple sclerosis, rickets, schizophrenia, autoimmune disease and several types of cancer [11–14]. Conversely, excessive UVR exposure frequently causes malignant skin diseases [11, 15] and mitochondrial (mtDNA) and nuclear (nDNA) damage [16–18]. Quantitative (mtDNA copy number) and qualitative (mutations) alterations in mtDNA in turn result in neurodegenerative and metabolic diseases [19] and amplifies immune suppression [20]. Although UVR exposure is the foremost cause of skin cancers, it is also a primary source of vitamin D in light-skinned individuals. As vitamin D protects against cancer and many other diseases, there is some disagreement concerning the health benefits and risks associated with UVR [8].

It has also been proposed that excessive UVR may have had influenced evolutionary processes, including extinction events such as the disappearance of megafauna during the late Pleistocene [21–23] and the extinction of some hominin species [24, 25]. It has been demonstrated that there is a correlation between hominin extinction events and the 41 ka orbital cycle attributable to change in our planets obliquity [25]. The first peaked 1.5 million years ago (mya) and closely followed the Olduvai (1.67 to 1.95 mya) and Gilsa (1.57 mya) geomagnetic excursions [26]. This event appears to coincide with the extinction of *Paranthropus robustus*, *P*. *bosei*, *Homo ergaster*, *H*. *rudolfensis* and *H*. *habilis* [25]. A second pulse of extinctions, affecting *H*. *neanderthalensis* and *H*. *floresiensis*, occurred during the last 120 ka and corresponds loosely with the Blake (138 ka to 100 ka) and Laschamp excursions [27–29]. The Laschamp excursion occurred at ~ 40 ka and has been cited as the most dramatic geomagnetic event to coincide with the extinction of *H*. *neanderthalensis* [24, 25, 30, 31]. Following the variable climate experienced during Marine Isotope Stage (MIS) 3 between 60 ka and 40 ka, the climate began to cool and by 30 ka Neanderthals were extinct. Proposed reasons for their demise include climate change, catastrophic natural events, disease epidemics, food shortages and the inability to compete with *H*. *sapiens* or other predatory species [32–39]. Because Neanderthals have been shown to have had lightly pigmented skin [40, 41], it has been suggested that increasing exposure to UVR may have been a factor in their disappearance [24, 25, 42].

These hypotheses are difficult to test because of the uncertainties concerning the dating of extinctions events and in calculating the impact of UVR changes at different latitudes and among different populations. Moreover, the absence of the MC1R gene variant (R307G) in Neanderthal and Denisovan genomes suggest that considerable variability in skin colour existed among archaic hominins, and that some Neanderthal and Denisovan groups had darker and therefore more UVR resistant skin tones [43, 44]. Fluctuations in UVR intensity may nevertheless have generated a selective pressure on human populations that could have affected the life expectancy of portions of those populations.

During the Laschamp excursion, the dipole geomagnetic moment (the intensity of the magnetic field currently expressed as 7.78 x 10^22^ A.m^2^), decreased by a factor of ~4 (from 10.5 x 10^22^ A.m^2^ at 49 ka to 2.6 x 10^22^ A.m^2^ at 41 ka), resulting in a twofold increase in cosmogenic nuclide deposition rates [45, 46]. At the same time, the amount of solar-derived UVR reaching earth’s surface increased by >25% [47, 48]. To put this surge in UVR into perspective, UVA and UVB exposure in sub-Saharan Africa currently exceeds 250 watts (W) per active area (m^2^) and rarely falls below 175 W/m^2^ [14]. UVR exposure levels >250 W/m^2^ present extreme exposure categories and are rated 10 (defined as posing a ‘very high risk of harm from unprotected sun exposure’) to 11+ (‘extreme risk of harm from unprotected sun exposure’) [49]. Exposure levels raised by 25%, such as that brought on by the Laschamp event (at >300 W/m^2^), would therefore have posed a sudden and severe health risk.

Strategies for dealing with increasing UVR exposure may have comprised both biological adaptations and behavioural advances [6, 50]. The use of complex forms of clothing may have presented one option in terms of limiting the adverse effects of excessive UVR. The ability to transform animal hide into leather is one of the most important technological skills mastered by humans [34] and genetic research suggests that this may have occurred between 107 ka and 72 ka [51, 52]. In lower latitudes characterised by high temperatures, clothing may have presented a disadvantage, such as inducing increasing perspiration and diminishing thermal insulation due to evaporative cooling. In such regions, the topical application of ochre may have presented an important novel behavioural innovation. Resembling the positive impact of technological innovation in enhancing human subsistence strategies [53–56], the topical application of ochre may therefore have served a significant function in terms of limiting the adverse effects of UVR exposure.

In this paper we recall the physiological mechanisms involved in the UVR protection and skin pigmentation, identify viable ethnographic candidates for the photoprotective use of red ochre, summarize the archaeological evidence attesting such a use in the past and, more importantly, experimentally test the efficacy of ochre as a topical photoprotective agent. As a result, we are able to demonstrate that certain types of iron-rich minerals, with or without binders, provide a perceptible degree of protection against UVR. We also evaluate the hypothesis that ochre may have been used as a sunscreen in the MSA and the Later Stone Age (LSA), discuss the advantages that such an innovation may have conveyed to our ancestors and propose a tentative scenario for its origin and geographic spread.

### UVR Protective Mechanisms

Human skin derives most of its pigmentation from two types of melanin, a brownish-black eumelanin and a reddish-yellow pheomelanin. Whereas constitutive skin colour is the genetically determined cutaneous melanin pigmentation generated without solar influence, facultative skin colour is the short-lived tanning reaction elicited by exposure to UVR [57]. Many theories have been proposed to explain the variation in human skin pigmentation. For the development of skin darkening, examples include the shielding of sweat glands and blood vessels [58], protection from cancer and the overproduction of vitamin D [59], guarding against folate photodestruction [60] and defense against microorganisms [61] and infection [62]. Hypotheses for skin lightening comprise sexual selection, adaptation to cold UVR deficient climates and the enhancement of vitamin D photoproduction [12]. An additional evolutionarily significant effect of UVR involves the metabolism of folate, a B vitamin essential for the modulation of gene expression and the production and repair of DNA [63]. Acute folate deficiencies result in male infertility and the increased prevalence of birth defects [64, 65]. Maintaining adequate levels of folate is essential for human reproduction, and the evolution of dark skin pigmentation was an effective means to achieve this [57, 66].

The most commonly accepted factor explaining the geographic distribution of human skin pigmentation is UVR exposure [63, 67]. Evidence for natural selection operating at low latitudes (establishing and maintaining dark pigmentation under high UVR conditions), and high latitudes (the development of light pigmentation under low UVR conditions) confirms that human skin coloration is a Darwinian adaptation, with the gradient of skin colours observed from the equator to the poles being the product of two clines operating over a spatially varying optimum of UVR distribution [68, 69]. The correlation between pigmentation in indigenous populations and latitude is therefore traceable to the correlation between skin colour and the intensity of UVR exposure [60, 65, 70–76] (Table 1).

**Table 1:** The classification of human skin types pertaining to responses to UVR exposure [70–75]. Constitutive values in brackets refer to the Von Luschan chromatic skin colour classification scale [76]. ‘Exposure’ refers to the amount of time during which UVR exposure would not induce perceptible damage to the skin. RSF is a factor characterizing the protective effect of a sunscreen against the generation of free radicals.

Skin type	Typical ethnicity	Constitutive skin color	Tanning ability	Sunburn susceptibility	Exposure (minutes)	Cancer susceptibility	Natural SPF	RSF factor	Recommended SPF
I	Celtic	White (1–5)	Very poor	High	5–10	High	1.0	<1.0	>30
II	Northern European, Scandinavian	White (6–10)	Poor	High	10–20	High	1.8	1.0	30
III	European, Caucasian	White (11–15)	Good	Moderate	20–30	Moderate	2.5	1.4	25
IV	Asian, Inuit, San Bushmen, Mediterranean	Olive (16–21)	Very good	Low	40–60	Low	3.5	1.8	15–10
V	Aboriginal, Papuan, Amerindian, Indian	Brown (22–28)	Very good	Very low	60	Very low	9.0	2.5	10
VI	African, Aboriginal, South Indian	Black (29–36)	Very good	Very low	>60	Very low	15.0	>2.5	<10

Differences in skin pigmentation do not result from differences in the number of melanocytes in the skin, but from differences in the melanogenic activity, the type of melanin produced in melanosomes and the size, number and packaging of melanosomes, with melanin content of melanosomes ranging from 17.9% to 72.3%. While melanin content in the epidermis differs by two-fold in Asian, and white skin, dark skin contains higher levels of melanin (3 to 6 fold), has more eumelanin and larger melanosomes than white skin. As a result, and whereas a dark epidermis allows only 7.4% of UVR-B and 17.5% of UVR-A to penetrate the skin, 24% UVR-B and 55% UVR-A passes through white skin [77].

That darker pigmentation conferred significant adaptive benefits is shown by the conservation of the MC1R, SLC45A2 and 70 other genetic loci involved in pigment production [78–80]. This indicates that there was strong evolutionary pressure to retain pigmentation among sub-Saharan Africa populations [62, 65]. Unlike SLC45A2, which occurs only amongst Europeans, SLC24A5, TYRP1 and KITLG are present at low frequencies in some sub-Saharan populations [80]. While these alleles may have arisen in and spread out of Africa with the early migrations of modern humans, many may derive from a series of admixture events with groups containing Eurasian genetic ancestry [81].

### Ethnographic Evidence

**Fig 1:**
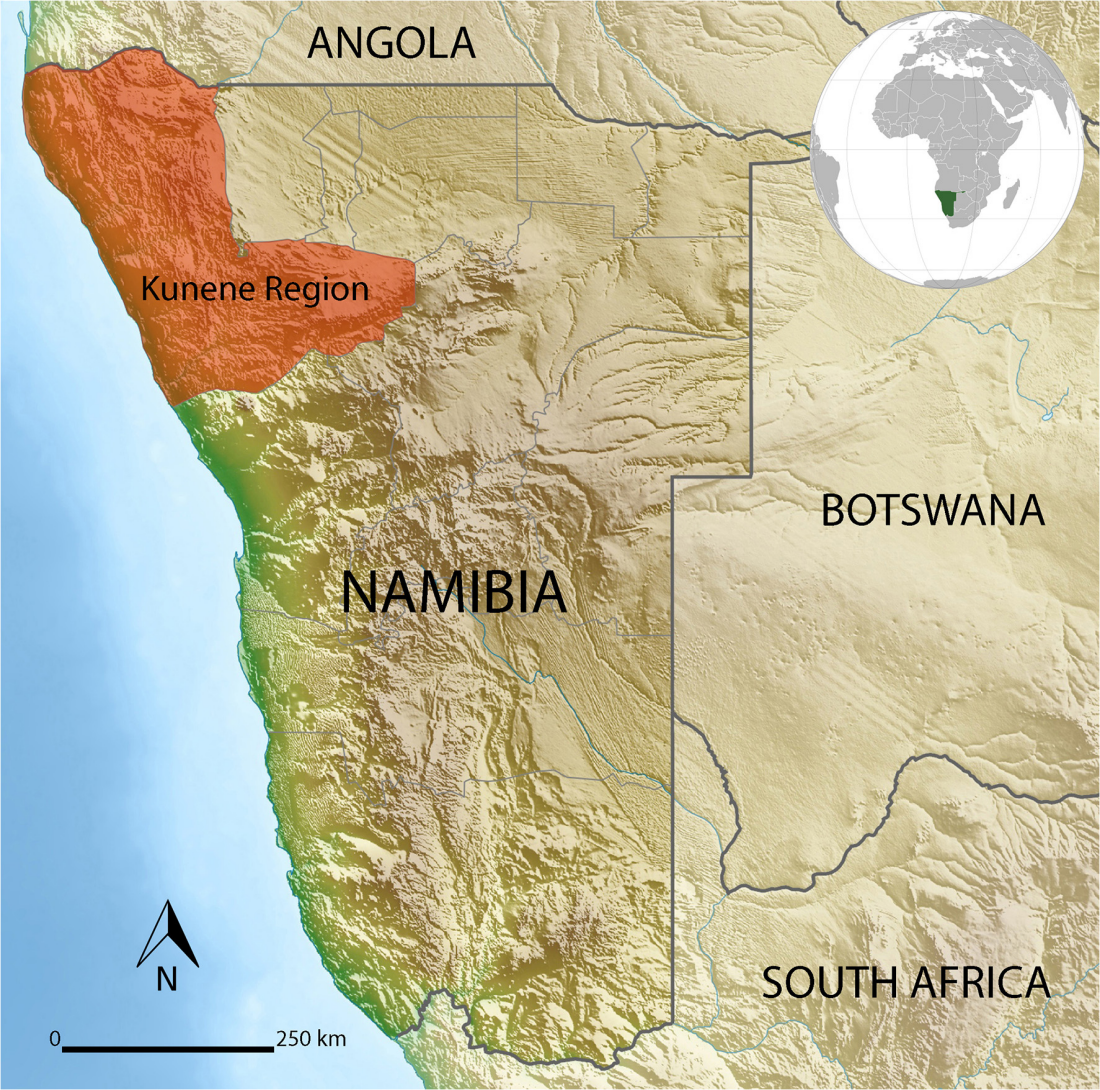
The location of the Kunene Region (red shaded area) in north-western Namibia (sourced from https://en.wikipedia.org/wiki/Template:Location_map_Namibia/doc).

Ethnographic accounts illustrating the use of red ochre as a cosmetic are abundant and have been reported for San hunter-gatherers [82–89], Khoe pastoralists [90], and Tswana [91] and Xhosa [92] agro-pastoralists. Foremost examples of the habitual use of red ochre as a body cosmetic comprise the Cushitic-speaking Hamar in Southern Ethiopia [93, 94] and the Ovahimba of Angola and Namibia [95–100]. The Ovahimba form part of a complex socio-economic assemblage of hunter-pastoralists who inhabit this region. They presumably represent a former Herero community who remained in the Kunene Region on a southward migration from the Central Lakes region into Namibia, or the final settlers in the terminal destination on a westward migration into the area after AD 1500 [99] (Fig 1). Recent archaeological excavations indicate that humans have inhabited the Kunene Region from at least 220 ka [101].

Ovahimba women are renowned for covering their bodies, hair and personal attire with a red ochre-based substance, ‘otjise’, which consists of equal parts of milk-derived clarified butter and red ochre powder [100]. Alexander (1838) observed that both women and men are ‘fond of greasing the skin’ with otjise [102], and personal observations and ethnographic interviews conducted by two of us (RFR, FDE) confirm this statement. The habitual application of otjise by men only declined during the 1960s, largely as a result of the presence of the South African Defense Force in the region and the subsequent employment of some Ovahimba as soldiers (Hepuite Venjakera, Nakara Tjimbosi and Tharhirwa Shawari pers. comm. 2012 [103, 104].

**Fig 2:**
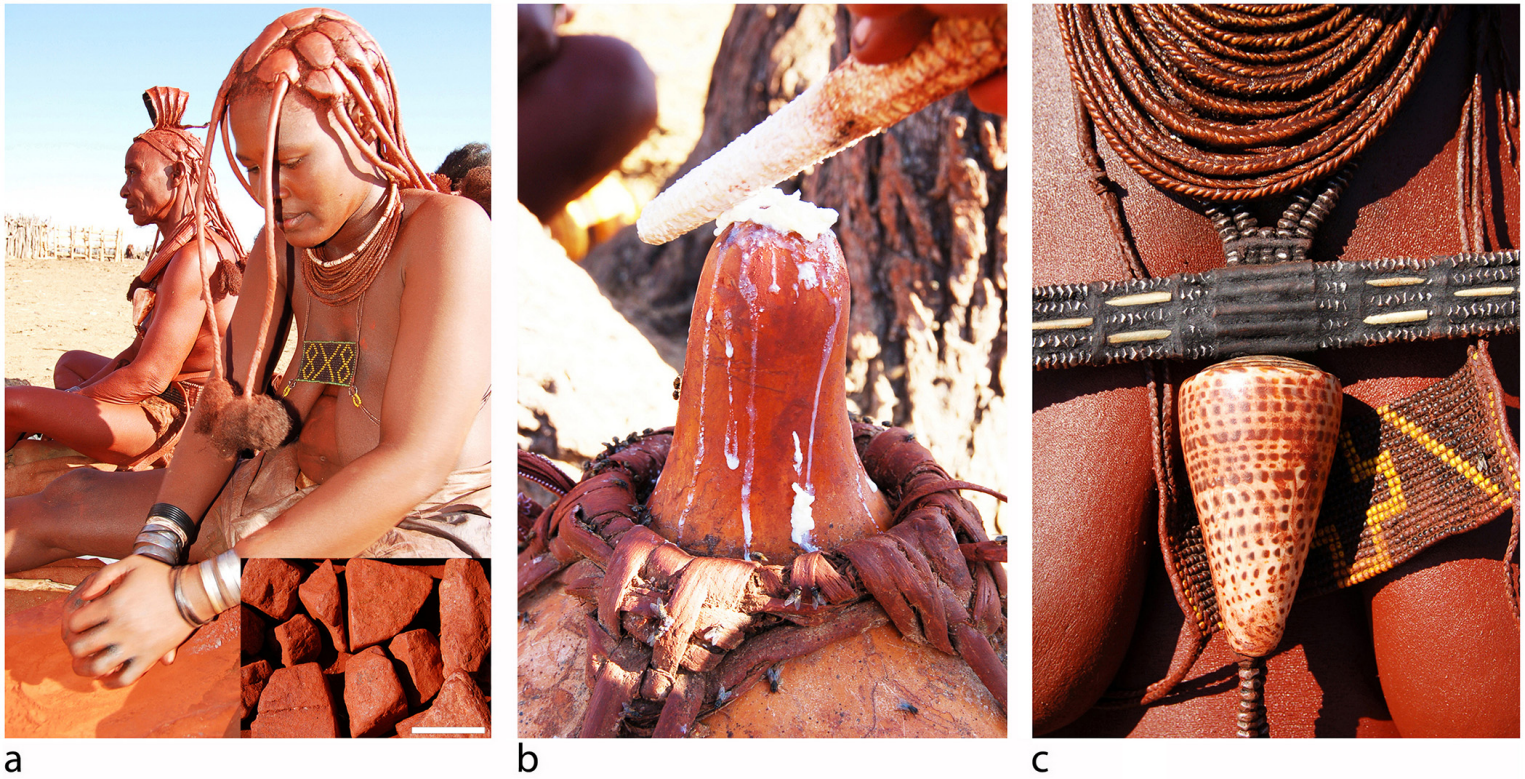
The production and application of otjise. (A) Red ochre powder is obtained by grinding chunks (inset—scale bar is 1cm) between a round upper and a flat lower grindstone, (B) after which it is mixed (at a 1:1 ratio between the palms) with milk-derived clarified butter and (C) applied to the hair, body and ornaments.

Clarified butter is produced by women and red ochre is mined or bought from Zemba land-owners or travelling merchants. While ochre processing occurs arbitrarily and forms part of the general sociable settings of daily life, the application of otjise occurs within the confines of women’s huts. Post menarche women mix roughly equal amounts of ochre powder and clarified butter together between the palms and apply 4.2mg of otjise per 1cm^2^ of skin surface. Women therefore require 60g of red ochre powder to cover their bodies (every 2 to 3 days) and 320g to treat their hair (every 2 to 4 weeks) (Fig 2) [100]. Otjise features in initiation ceremonies, is applied by men when they are to be wed or when they undertake extensive journeys and used to cover human corpses prior to interment [95, 97]. Personal belongings such as leather headdresses and aprons, wooden containers and jewellery are also covered with the compound, apparently for both symbolic and functional (preservative) purposes. Whereas infants are intentionally covered with the mixture, regular contact between women and children result in the substantial transfer of the red compound to toddlers. Ethnographic interviews conducted by two of us (RFR, FDE) (refer to http://www.canal-u.tv/video/cerimes/the_color_of_the_ovahimba.15970) revealed that, besides the intrinsic symbolic significance of red ochre, it also fulfills several secondary functional roles, including that as a topical sun-protection element.

### Archaeological Evidence

The exploitation of ochre of variable colour and geological origin is not restricted to *Homo sapiens* [105–107] and it does not reflect a sudden change signifying a shift away from archaic to modern and symbolic patterns of behaviour. Red ochre residues have been found inside perforated shell beads from African and Near Eastern MSA and Levantine Mousterian sites dated from 92 ka to 60 ka [108–112]. Since strung beads were in all probability worn around the neck or wrists [113], these residues most likely transferred from human bodies onto the shells. But as direct evidence on how and why ochre was utilised during the MSA and LSA is not available, diverse interpretations for its usage have been proposed. Although the symbolic and functional significance of ochre is a hotly debated topic [114–123], these uses are not necessarily mutually exclusive.

Cultural innovations seeking to reduce the impact of UVR, such as the habitual topical application of powdered ochre, could also have reduced the selective pressure induced by increasing exposure to UVR. Changes in climate and ecology have been shown to provoke speciation and extinction [25, 124–127] and it has been proposed that these may have acted as selective pressures for enhanced human cognition [128–131]. MIS 5e to 5d represent the most dynamic periods of innovation and may have generated the conditions for the expansion of populations within and out of Africa [4]. Examples of geographically confined cultural traditions that may represent the first instances of socially mediated symbolic responses to changing ecological conditions, include abstract engraved designs on ochre [132–135] and perforated marine shell beads [106, 108–113]. It has been shown that cultural niche construction [136] played a key role in positively influencing human evolutionary outcomes [137, 138]. Given that dark-skinned individuals are also subject to the harmful effects of UVR [68, 69], the development of effective sun-protection strategies was essential. One solution may have entailed the topical application of organic or inorganic agents perceived to reduce the detrimental effects of UVR.

## Materials and Methods

Permission to carry out the Namibian ethnographic research was provided by the Namibian Ministry of Culture and the Namibian Filming Authority. All individuals referred to and represented in this manuscript have given written informed consent to publish case details, including data and photographs. Permission to collect ochre samples from the Palaeozoic Bokkeveld Group deposits was provided by private landowners. Informed consent for *in vivo* SPF experiments was obtained in written form and approved by the Photobiology Laboratory of Medunsa Campus of the University of Limpopo (Sefako Makgatho University of Health Sciences) Research and Ethics Committee (Project Number MREC/H/158/2014). One of the authors (RFR) participated in the *in vivo* SPF trials.

### Ochre

The *in vivo* experimental results reported here follows from an initial *in vitro* SPF assessment of 24 ochre samples derived from ethnographic sources and geological resources likely to have been exploited during the MSA [100, 139]. Samples one to six derive from Okamanga, Ovinjange and Otjongoro villages in the Kunene Region of Northern Namibia. These were acquired by Ovahimba women in Opuwo and derive from Ombumbuu red ochre mines near Ruacana. Red ochre derived this geological source appears to be particularly sought after by Ovahimba women across the Kunene Region. The samples were processed into powder by Ovahimba women by crushing and grinding ochre chunks between a round upper and a flat lower grindstone. Samples 7 to 12 were acquired in Opuwo and were experimentally processed by direct grinding onto a coarse quartzite surface. This method resembles the techniques used to produce ochre powder during the MSA [121]. An additional twelve samples, comprising yellow, grey and red specimens, were collected from the Palaeozoic Bokkeveld Group deposits of the Cape Supergroup [140]. These are mostly fissile shales and comprise the most likely source of ochre at Blombos Cave [133]. Six samples were processed into powder by direct grinding onto a coarse quartzitic sandstone surface, and six by way of the technique employed by Ovahimba women. Table 2 presents information concerning the samples subjected to UVR during the experiments.

**Table 2:** Experimental ochre samples subjected to SPF, ED-XRF, XRD, granulometric, colorimetric and UVR analyses.

Sample	Source	Description	Processing method
1	Okamanga	Fine ground ochre powder	Ethnographically ground (by Ovahimba)
2	Okamanga	Fine ground ochre powder	Ethnographically ground (by Ovahimba)
3	Okamanga	Fine ground ochre powder	Ethnographically ground (by Ovahimba)
4	Okamanga	Fine ground ochre powder	Ethnographically ground (by Ovahimba)
5	Ovinjange	Fine ground ochre powder	Ethnographically ground (by Ovahimba)
6	Otjongoro	Fine ground ochre powder	Ethnographically ground (by Ovahimba)
7	Opuwo	Fine-grained red ochre chunk	Experimentally ground onto quartzite slab
8	Opuwo	Fine-grained red ochre chunk	Experimentally ground onto quartzite slab
9	Opuwo	Fine-grained red ochre chunk	Experimentally ground onto quartzite slab
10	Opuwo	Fine-grained red ochre chunk	Experimentally ground onto quartzite slab
11	Opuwo	Fine-grained red ochre chunk	Experimentally ground onto quartzite slab
12	Opuwo	Fine-grained red ochre chunk	Experimentally ground onto quartzite slab
13	Napier	Soft yellow limonite chunk	Experimentally ground onto quartzite slab
14	Blombos	Soft grey shale cobble	Experimentally ground onto quartzite slab
15	Napier	Medium hard red shale chunk	Experimentally ground onto quartzite slab
16	Napier	Medium hard red shale chunk	Experimentally ground onto quartzite slab
17	De Hoop	Soft light red shale chunk	Experimentally ground onto quartzite slab
18	Cape Point	Hard red shale ochre chunk	Experimentally ground onto quartzite slab
19	Napier	Soft yellow limonite chunk	Experimentally ground (like Ovahimba)
20	Blombos	Soft grey shale cobble	Experimentally ground (like Ovahimba)
21	Napier	Medium hard red shale chunk	Experimentally ground (like Ovahimba)
22	Napier	Medium hard red shale chunk	Experimentally ground (like Ovahimba)
23	De Hoop	Soft light red shale chunk	Experimentally ground (like Ovahimba)
24	Cape Point	Hard red shale ochre chunk	Experimentally ground (like Ovahimba)

### Binders

For the SPF assessments, ochre samples were mixed (at a 1:1 ratio) with two types of organic binders. Clarified butter was procured from Otjongoro village and subcutaneous fat from a kudu (*Tragelaphus strepsiceros*) antelope. As per the SANS 1557/ISO 24444 *in vivo* SPF testing protocol [6], we applied 2mg of experimental materials per 1cm^2^ of skin surface during the experiments. Although Ovahimba women typically apply 4.2mg otjise per cm^2^, we adhered to the standard 2mg/cm^2^ ratio to acquire comparable SPF values. Given that Ovahimba women apply an additional 2.2mg per cm^2^, the actual SPF values are likely to be significantly higher than the results presented here.

### Analytical methods

#### Ultraviolet radiation (UVR)

The assessment of the efficiency of a sunscreen is based on the value of its ‘sun protection factor’ (SPF), which reflects the degree of protection against UVR-induced erythema. At a certain exposure dose of UVB, minimally detectable redness can be observed. This is referred to as the minimal erythema dose (MED). SPF is the ratio of MED after sunscreen application and baseline (untreated) MED. Whereas SPF values generally denote the efficiency of a sunscreen to protect the skin from medium-wave UVB (280–315 nm) radiation, UVA is also a component of the solar simulated radiation which forms part of the *in vivo* SPF test. UVAPF denotes its UVA or long-wave ultraviolet (315–400 nm) protection factor. A sunscreen with an SPF of 2 filters out 50% of UV, an SPF of 15 filters out 93% UV and an SPF of 50 filters out 98% UV [6, 141]. In theory, the application of a product with SPF of 5 provides sun protection for five times longer than unprotected skin. Modern sunscreens typically comprise UVR absorbing chemicals that attenuate the amount of UVR reaching viable cells in the skin. Sunscreens contain chemical filters that absorb UVB radiation and physical filters that attenuate UVA and UVB radiation through reflection and scattering [6, 50]. Whereas organic molecules are selected for their UVR absorption capacity (cinnamates, benzophenone and butylmethoxy dibenzoyl methane), inorganic particles (zinc, titanium and iron oxides) are selected for their capacity to absorb, reflect or scatter UVR [142]. Sunscreens do not completely prevent photodamage but they do alter the UVR spectrum that reaches skin cells [7].

The UVR protection capacities (SPF and UVAPF values) of ochre samples were established by means of a series of initial *in vitro* and *in vivo* experiments performed under controlled conditions at the Photobiology Laboratory, Medunsa Campus (Sefako Makgatho University), South Africa [143]. As an initial screening method, experimental samples were subjected to *in vitro* protection tests [100]. The samples were applied to Transpore tape at a ratio of 2mg/cm^2^ and analysed according to the SANS 1557:1992 procedure. Critical Wavelength (CW) was determined using the Optometrics SPF 290 method. This method was chosen as there is unlikely to be photodegradation of the samples, hence the more recent ISO/SANS 24443 were not considered necessary. The CW is the wavelength below which 90% of the UV protection is situated. Accordingly, the higher the CW, the higher the UVA protection ranges of the products. Following the initial *in vitro* experiments, the six ochre samples with the highest SPF values (red ochre samples 1, 5, 7, 10, 16 and 18) were selected for *in vivo* SPF assessment. Informed consent was obtained from all research participants and the principles of the Declaration of Helsinki were strictly adhered to. The *in vivo* SPF test method followed the SANS 1557/ISO 24444 SPF test protocol. Tests were performed on three test subjects, designated A, B and C, with skin phototype II, and conducted using an application quantity of 2mg/cm^2^. The UV irradiation sources consisted of a calibrated Multiport Solar Simulator. An SPF 15 reference standard was included in the test and erythema was assessed visually by two experienced assessors 24 hours after irradiation.

#### Visible spectroscopy

To determine whether colour played a role in the UVR reflectance and absorption capabilities of ochre, visible spectroscopy was employed to obtain L*a*b* values for each sample. We used an Avantes AvaSpec2048 fibre optic spectrometer equipped with a 2048 pixel CCD detector set to operate in the retrodiffusion mode. This instrument is equipped with an optical fibre probe, which is set in contact with the powder samples stored in transparent plastic bags. An AvaLight-HAL was used as an illumination source. The equipment is calibrated with a Halon D65 white reference sample in the same lighting conditions as for the archaeological samples. The colour parameters were obtained by Avasoft 7.5 software.

#### Particle size analyses

Since particle size distribution may play an important role in determining the UVR protection capacities of clay minerals [144], ochre samples were subjected to particle size analyses. We used a Horiba LA950 (0.01μm—3000μm) laser micro-granulometer and analysed the data with NextSpec 7.10 software. The Mie solution to Maxwell’s equations provides the basis for measuring the size of particles through the scattering of electromagnetic radiation [145]. We employed this solution to calculate the particle size distribution in aqueous solution (refractive index 1,333). For sediments not predominantly comprised of iron oxides and hydroxides, refractive indices for the Mie calculation were based on the standard sediment refractive index of 1.55i–0.01i. For the samples consisting primarily of hematite, calculations were made with a refractive index of 2.94i–0.01i. The pre-treatment of samples included suspension in sodium hexametaphosphate (5 g/L) for 12 hours and 60 seconds of ultrasonification in the micro-granulometer to achieve optimal dispersion. The results are expressed through the use of D10, D25 and D50 values, the size of the particles that represent the 10th, 25th and the 50th percentile of the cumulative granulometric distribution. A sample with a D10 value of 15μm is a sample in which 10% of the particles are smaller or equal to 15μm. For the ternary plots, standard geological scales and terminologies were employed. Clay, silt and sand categories comprise the amounts of particles smaller than 7μm, between 7μm and 63μm and between 63μm and 2mm, respectively. The limit of 7μm is based on the demonstration by Konert and Vandenberghe (1997) [146] that this is the value that correlates better, when laser granulometry is used, to the particle size value of the clay fraction (<2μm) of previous sedimentological methods.

#### Energy dispersive X-ray fluorescence (ED-XRF)

The ED-XRF measurements were carried out using a portable SPECTRO xSORT X-ray fluorescence spectrometer from AMETEK equipped with a silicon drift detector (SDD) and a low power W X-ray tube with an excitation source of 40 kV. Samples were positioned above a 7mm diameter spectrometer aperture and analysed from below for 60 seconds. For data treatment, we used the peak count rates of all detected elements and quantitative data for a selection of elements. The quantitative data were calculated according to a calibration operated with AMETEK X-LabPro software. This calibration was constructed by using 12 certified and local references and allows for the semi-quantification of 7 major and trace elements among the most abundant in ferruginous rocks (normalised concentrations are presented in oxide weight) [147].

#### X-ray diffraction (XRD)

Ochre samples were analysed by way of X-ray diffraction using a silicon calibrated Panalytical diffractometer with a Bragg-Brentano (theta-theta) configuration. Data acquisition times of 30 minutes allowed an angle range of 8 to 80° with a resolution of 0.02°. The diffraction spectra were analysed using EVA application software coupled with the JCPDS-ICDD database. The mineral species were identified by their respective combinations of two or three characteristic peaks. This first qualitative determination was validated and quantified using TOPAS software. The best fit for each measure was determined using Rietveld’s refinement method [148]. This procedure ensured that the determinations made with EVA did not overlook information masked by overlapping peaks and allowed precise quantification of the relative proportions of each mineral.

## Results

### Characterisation of ochre samples

#### Colourimetry

The colorimetric values of samples are presented in Table 3. Pigment powder produced by Ovahimba women appear colorimetrically homogeneous and display differences in L*a*b* coordinates (ΔE) of less than 2.4. This is well below 5, the value generally considered as the limit above which a significant colour difference can be perceived by humans [149]. The South African Bokkeveld Group red ochre samples display the same range of hue and chroma (similar a* and b* values) as the other red ochre samples. Only lightness (L*) values are dissimilar, with Ovahimba red ochres being darker.

**Table 3:** L*a*b* colourimetric properties of ethnographic and experimental ochre samples with L* representing lightness (100 white / 0 black), a* indicating variations between red (+) and green (-), and b* representing variations between yellow (+) and blue components (-). The first sample was arbitrarily selected as the reference sample and the Delta E (ΔE) values calculated accordingly.

Sample	Source	Processing method	Colour	L*	a*	b*	Delta E
1	Okamanga	EGO	Dark red	39	32	30	-
2	Okamanga	EGO	Dark red	37	29	28	4.1
3	Okamanga	EGO	Dark red	40	30	28	3.0
4	Okamanga	EGO	Dark red	37	29	28	4.1
5	Ovinjange	EGO	Dark red	40	32	30	1.0
6	Otjongoro	EGO	Dark red	40	31	29	1.7
7	Opuwo	GQS	Dark red	39	31	29	1.4
8	Opuwo	GQS	Dark red	41	32	31	1.2
9	Opuwo	GQS	Dark red	40	32	30	1.0
10	Opuwo	GQS	Dark red	39	31	29	1.4
11	Opuwo	GQS	Dark red	41	29	28	1.1
12	Opuwo	GQS	Dark red	46	24	21	13.9
13	Napier	GQS	Yellow	81	9	43	49.6
14	Blombos	GQS	Light grey	88	-1.5	8	63.6
15	Napier	GQS	Light red	47	30	30	8.2
16	Napier	GQS	Maroon	52	28	28	18.7
17	De Hoop	GQS	Dark red	59	24	25	22.1
18	Cape Point	GQS	Orange-red	47	33	35	9.5
19	Napier	EGLO	Yellow	71	11	43	40.4
20	Blombos	EGLO	Light grey	84	-1	8	60.0
21	Napier	EGLO	Light red	46	24	21	13.9
22	Napier	EGLO	Maroon	51	22	18	19.7
23	De Hoop	EGLO	Dark red	56	19	18	24.5
24	Cape Point	EGLO	Orange-red	42	30	30	3.6

EGO: ground by Ovahimba; GQS: Experimentally ground onto quartzite slab; EGLO: Experimentally ground (like Ovahimba)

It has been shown that different ochre processing techniques result in differences in pigment powder consistency and colour [121]. In this study, ochre powder produced by direct grinding presents higher chroma (a* and b* values) than powder obtained by grinding with lower and upper grindstones. Noticeable differences in colour resulting from different processing techniques are exhibited by the Napier 15 (ground directly onto a coarse quartzite surface as evidenced by examples derived from MSA and LSA contexts) and 21 (ground conventionally between an upper and lower grindstone such as practiced by the Ovahimba) and the Napier 16 (direct) and 22 (conventional) samples.

#### Granulometry

**Fig 3:**
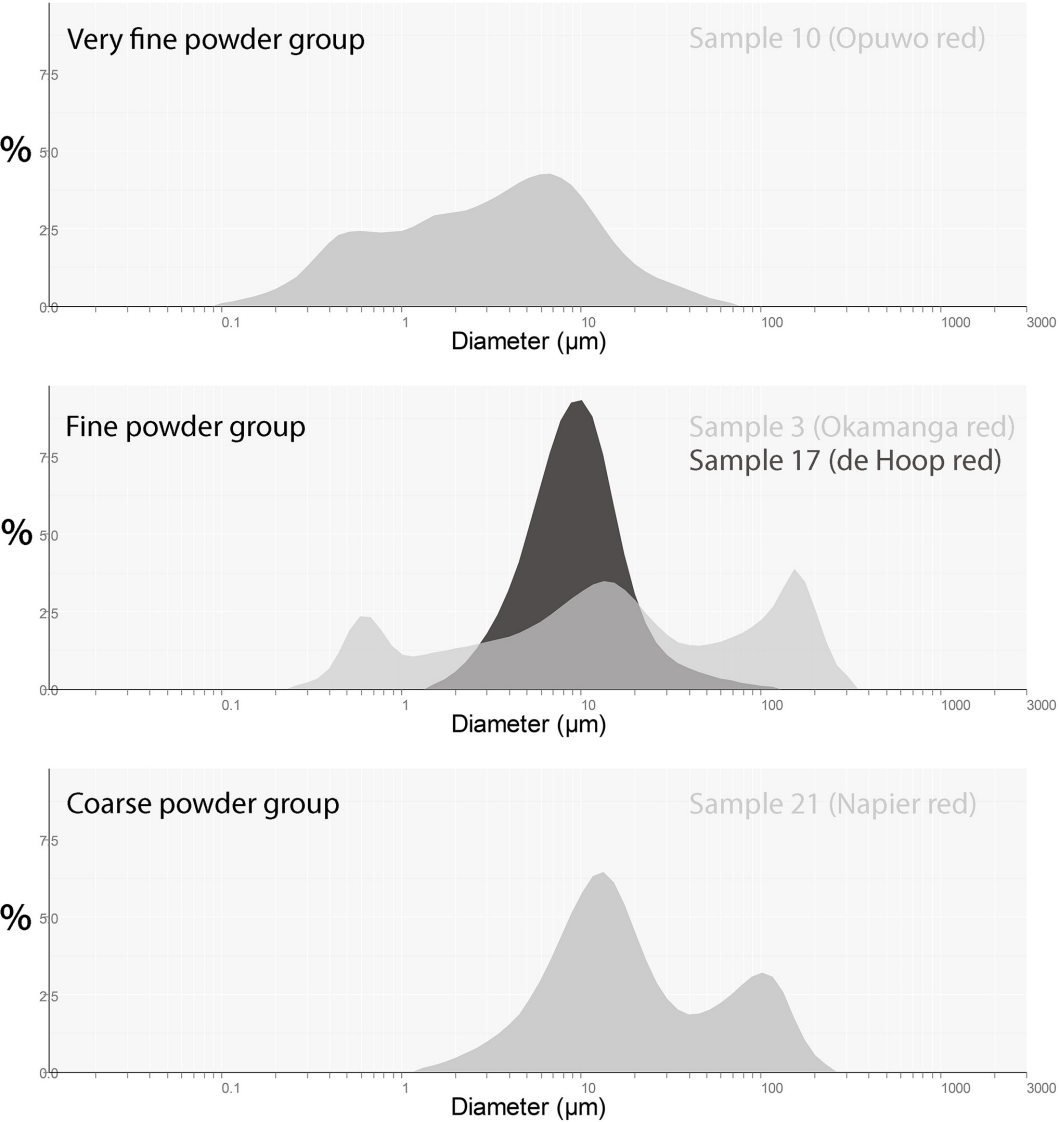
Representative grain size distributions for each group.

Grain size analysis identifies three broad groups of samples including 1) very fine powders, 2) fine powders and 3) coarse powders (Table 4). The first group has a D10 value close to 1μm and a clay-size proportion approximating or exceeding 60%. This is the case for the samples from Opuwo which were ground directly onto quartzite surfaces. The fine powders display D10 values ranging between 1.5μm and 4μm and a clay content <50%. Most of the samples processed by Ovahimba women fall into this category. The third group is characterised by a D10 close to 5 or higher and a clay-sized content <30%. The samples experimentally processed by way of the technique employed by the Ovahimba belong to this group. Differences in grain size between samples ground by the Ovahimba and experimentally by the authors are significant. Results suggest that, in order to be effective, the Ovahimba technique must be applied to fine-grained iron-rich sources. The application of this technique to process ochre with a high proportion of quartz, such as those from Cape Point, produces coarse-grained pigment powder (Table 4). Every sample shows multimodal grain size distribution (Fig 3) which is classical in grinding processes and especially in these materials comprised of multiple mineral phases of dissimilar hardness.

**Table 4:** Results for particle size analyses of ochre samples.

Sample	Source	Processing method	Refractive index	D10 (μm)	D25 (μm)	D50 (μm)	D75 (μm)	D90 (μm)	Clay (%)	Silt (%)	Sand (%)	Group
1	Okamanga	EGO	Hematite	1.1	2.7	6.5	14.7	39.8	52	44	4	Very fine powder
2	Okamanga	EGO	Hematite	1.8	4.4	10.9	51.5	98.0	37	42	21	Fine powder
3	Okamanga	EGO	Hematite	1.8	4.7	11.5	74.0	142.3	35	37	28	Fine powder
4	Okamanga	EGO	Hematite	1.7	4.0	9.6	41.3	83.8	40	43	17	Fine powder
5	Ovinjange	EGO	Hematite	0.6	1.5	3.9	8.6	30.5	69	27	3	Very fine powder
6	Otjongoro	EGO	Hematite	1.5	4.0	9.5	36.4	108.4	40	41	19	Fine powder
7	Opuwo	GQS	Hematite	0.8	2.0	4.0	6.6	9.6	78	22	0	Very fine powder
8	Opuwo	GQS	Hematite	0.8	1.9	4.1	7.1	11.4	74	25	1	Very fine powder
9	Opuwo	GQS	Hematite	1.0	2.3	4.5	7.2	10.3	74	26	0	Very fine powder
10	Opuwo	GQS	Hematite	0.4	1.0	3.1	7.2	13.2	74	26	0	Very fine powder
11	Opuwo	GQS	Sediment	0.7	3.4	6.1	10.0	16.8	57	41	1	Very fine powder
12	Napier	GQS	Sediment	1.8	3.4	5.7	9.3	20.7	62	33	5	Very fine powder
13	Blombos	GQS	Sediment	3.5	5.1	7.7	11.4	18.1	44	54	2	Fine powder
14	Napier	GQS	Sediment	5.1	7.0	9.9	15.4	64.1	25	65	10	Coarse powder
15	Napier	GQS	Sediment	2.0	4.6	8.6	14.2	22.5	40	59	1	Fine powder
16	De Hoop	GQS	Sediment	2.2	4.7	9.4	16.9	36.9	38	58	5	Fine powder
17	Cape Point	GQS	Sediment	3.8	5.7	8.6	12.6	18.8	37	62	1	Fine powder
19	Napier	EGLO	Sediment	4.7	7.6	13.2	41.3	101.1	22	60	19	Coarse powder
20	Blombos	EGLO	Sediment	6.0	8.3	12.7	58.5	125.6	16	60	24	Coarse powder
21	Napier	EGLO	Sediment	5.0	8.5	14.9	44.4	97.8	18	63	19	Coarse powder
22	Napier	EGLO	Sediment	5.3	9.3	16.3	44.1	102.3	16	65	19	Coarse powder
23	De Hoop	EGLO	Sediment	5.5	8.4	13.7	33.3	81.1	17	68	15	Coarse powder
24	Cape Point	EGLO	Hematite	2.4	6.1	17.0	76.0	128.4	28	42	30	Fine powder

EGO: ground by Ovahimba; GQS: Experimentally ground onto quartzite slab; EGLO: Experimentally ground (like Ovahimba)

#### Mineralogical composition

**Fig 4:**
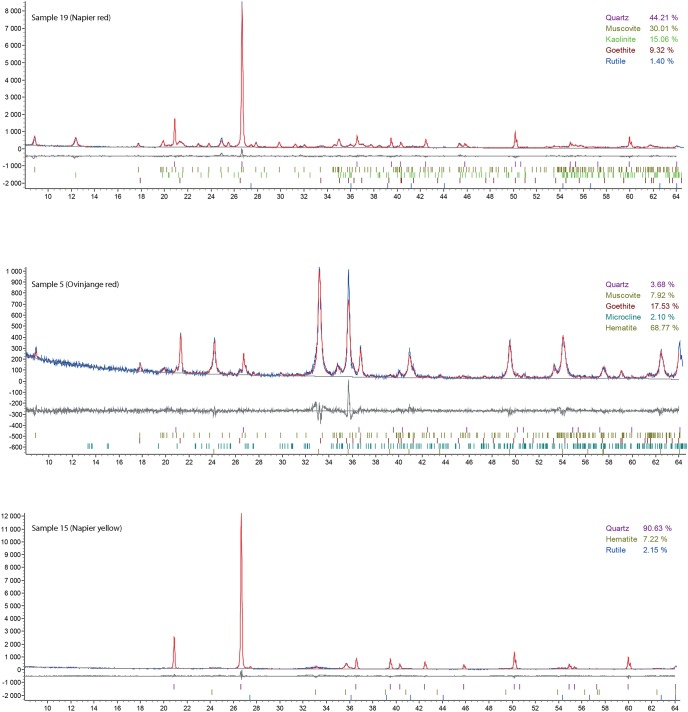
XRD spectra recorded for ochre samples 5 (Ovinjange fine ground ochre powder), 15 (Napier medium hard red shale chunk) and 19 (Napier soft yellow limonite chunk).

Analysed samples comprise variable amounts of iron oxides, clays and quartz (Table 5). Goethite and hematite are present in almost all samples, with hematite being the primary constituent. No hematite is detected in the yellow (13 and 19) and grey ochre samples (14 and 20), the former containing only goethite and the latter being the only sample in which iron oxide could not be detected. In comparison with other red samples, Ovahimba red ochre typically contains less quartz and clay minerals (illite and kaolinite) and more iron oxides (Fig 4). Most of the Ovahimba samples are composed of more than 60% iron oxides/oxy-hydroxides. In the samples derived from Cape Point, the concentration in iron oxide/oxy-hydroxide does not exceed 20%.

**Table 5:** Results of XRD analyses of ethnographic and experimental ochre samples.

Sample	Source	Processing method	Hematite %	Goethite %	Quartz %	Illite %	Kaolinite %	Feldspar %	Dolomite %	Halite %	Rutile %	Total %
1	Okamanga	EGO	55.89	11.04	9.02	16.80	nd	7.25	nd	nd	nd	100.00
2	Okamanga	EGO	55.82	10.13	17.11	10.77	nd	6.16	nd	nd	nd	99.99
3	Okamanga	EGO	48.20	10.33	20.43	13.60	nd	7.44	nd	nd	nd	100.00
4	Okamanga	EGO	53.46	10.61	18.87	10.27	nd	6.79	nd	nd	nd	100.00
5	Ovinjange	EGO	67.81	17.39	3.51	9.32	nd	1.98	nd	nd	nd	100.01
6	Otjongoro	EGO	52.64	10.25	10.28	18.03	nd	8.80	nd	nd	nd	100.00
7	Opuwo	GQS	57.04	10.01	9.19	16.91	nd	6.85	nd	nd	nd	100.00
8	Opuwo	GQS	56.25	14.48	7.40	15.74	nd	6.13	nd	nd	nd	100.00
9	Opuwo	GQS	64.02	14.87	6.07	9.92	nd	5.13	nd	nd	nd	100.01
10	Opuwo	GQS	65.07	17.64	4.96	10.15	nd	2.19	nd	nd	nd	100.01
11	Opuwo	GQS	29.61	3.37	16.63	29.85	nd	16.78	3.75	nd	nd	99.99
12	Opuwo	GQS	28.41	1.75	8.45	42.21	nd	16.88	2.30	nd	nd	100.00
13	Napier	GQS	nd	10.78	35.91	35.48	14.42	nd	nd	1.88	1.53	100.00
14	Blombos	GQS	nd	nd	29.03	66.80	2.21	nd	nd	0.71	1.25	100.00
15	Napier	GQS	9.15	nd	88.85	nd	nd	nd	nd	nd	2.00	100.00
16	Napier	GQS	9.34	nd	75.95	nd	13.67	nd	nd	nd	1.04	100.00
17	De Hoop	GQS	2.28	nd	56.80	29.28	9.43	nd	nd	1.14	1.07	100.00
18	Cape Point	GQS	19.60	nd	34.65	nd	44.29	nd	nd	nd	1.46	100.00
19	Napier	EGLO	nd	9.32	44.21	30.01	15.06	nd	nd	nd	1.40	100.00
20	Blombos	EGLO	nd	nd	28.48	67.11	3.25	nd	nd	nd	1.15	99.99
21	Napier	EGLO	7.46	nd	91.28	nd	nd	nd	nd	nd	1.25	99.99
22	Napier	EGLO	6.80	nd	83.23	nd	8.83	nd	nd	nd	1.13	99.99
23	De Hoop	EGLO	1.45	nd	59.31	26.12	11.13	nd	nd	0.79	1.20	100.00
24	Cape Point	EGLO	19.60	nd	34.65	nd	44.29	nd	nd	nd	1.46	100.00

EGO: ground by Ovahimba; GQS: Experimentally ground onto quartzite slab; EGLO: Experimentally ground (like Ovahimba); nd: not detected

#### Elemental composition

Fe and Si are the two major elements detected by ED-XRF (Table 6). The relative contents of Fe (Fe_2_O_3_), Si (SiO_2_) and other minor elements were calculated following calibration and normalization to 100%. CaO and K_2_O are only present as minor components, except in the sample from Blombos (sample 14) which contain higher proportions of K. TiO_2_ is also present as a minor constituent. Compared to samples collected from the Bokkeveld shales, the ratio of Fe_2_O_3_ to SiO_2_ is significantly higher in most of the Ovahimba samples. Two samples from Opuwo (11 and 12) present lower Fe contents and lower ratio of Fe_2_O_3_ to SiO_2_ than the other Ovahimba samples. This is consistent with the XRD results. Only the samples from Cape Point (18 and 24) present Fe_2_O_3_ to SiO_2_ ratios as high as those recorded for the Opuwo samples.

**Table 6:** Results of ED-XRF analyses of ethnographic and experimental ochre samples.

Sample	Source	Processing method	SiO_2_	Error (%)	Fe_2_O_3_	Error (%)	MnO	Error (%)	K_2_O	Error (%)	CaO	Error (%)	TiO_2_	Error (%)	V_2_O_3_	Error (%)	Total
1	Okamanga	EGO	27.45	0.93	67.35	0.02	0.06	0.00	2.83	0.02	0.72	0.01	1.45	0.01	0.14	0.00	100.00
2	Okamanga	EGO	29.30	1.02	64.60	0.03	0.06	0.00	2.10	0.02	2.67	0.02	1.13	0.01	0.14	0.00	100.00
3	Okamanga	EGO	34.94	1.18	60.75	0.01	0.07	0.00	2.36	0.02	0.49	0.01	1.26	0.01	0.13	0.00	100.00
4	Okamanga	EGO	31.21	1.00	62.62	0.03	0.07	0.00	2.05	0.02	2.85	0.02	1.06	0.01	0.13	0.00	100.00
5	Ovinjange	EGO	15.08	0.68	81.10	0.02	0.07	0.00	1.78	0.02	0.39	0.01	1.39	0.01	0.19	0.00	100.00
6	Otjongoro	EGO	34.60	0.99	60.31	0.02	0.02	0.00	2.68	0.02	0.91	0.01	1.35	0.01	0.13	0.00	100.00
7	Opuwo	GQS	33.72	0.98	61.36	0.02	0.05	0.00	2.99	0.02	0.47	0.01	1.27	0.01	0.14	0.00	100.00
8	Opuwo	GQS	26.89	0.92	68.20	0.02	0.05	0.00	2.55	0.02	0.72	0.01	1.45	0.01	0.14	0.00	100.00
9	Opuwo	GQS	19.81	0.77	76.16	0.02	0.04	0.00	1.99	0.02	0.53	0.01	1.31	0.01	0.17	0.00	100.00
10	Opuwo	GQS	9.80	0.52	85.75	0.02	0.06	0.00	1.65	0.02	1.24	0.01	1.29	0.01	0.21	0.00	100.00
11	Opuwo	GQS	55.59	1.26	36.53	0.01	0.02	0.00	4.83	0.03	1.95	0.01	0.99	0.01	0.08	0.00	100.00
12	Opuwo	GQS	61.16	1.23	29.73	0.02	0.02	0.00	6.23	0.03	1.42	0.01	1.33	0.01	0.09	0.00	100.00
13	Napier	GQS	78.30	1.36	15.69	0.01	0.01	0.00	4.61	0.02	nd	nd	1.34	0.01	0.06	0.00	100.00
14	Blombos	GQS	75.14	1.30	5.46	0.01	0.04	0.00	17.08	0.06	nd	nd	2.17	0.01	0.10	0.01	100.00
15	Napier	GQS	76.97	0.93	21.76	0.01	nd	nd	nd	nd	nd	nd	1.23	0.01	0.04	0.00	100.00
16	Napier	GQS	81.49	1.04	17.75	0.01	nd	nd	nd	nd	nd	nd	0.72	0.01	0.04	0.00	100.00
17	De Hoop	GQS	84.63	1.27	11.27	0.01	0.02	0.00	3.19	0.02	nd	nd	0.84	0.01	0.05	0.00	100.00
18	Cape Point	GQS	57.71	1.40	40.96	0.02	nd	nd	nd	nd	nd	nd	1.29	0.01	0.04	0.03	100.00
19	Napier	EGLO	78.30	1.36	15.69	0.01	0.01	0.00	4.61	0.02	nd	nd	1.34	0.01	0.06	0.00	100.00
20	Blombos	EGLO	75.14	1.30	5.46	0.01	0.04	0.00	17.08	0.06	nd	nd	2.17	0.01	0.10	0.01	100.00
21	Napier	EGLO	76.97	0.93	21.76	0.01	nd	nd	nd	nd	nd	nd	1.23	0.01	0.04	0.00	100.00
22	Napier	EGLO	81.49	1.04	17.75	0.01	nd	nd	nd	nd	nd	nd	0.72	0.01	0.04	0.00	100.00
23	De Hoop	EGLO	84.63	1.27	11.27	0.01	0.02	0.00	3.19	0.02	nd	nd	0.84	0.01	0.05	0.00	100.00
24	Cape Point	EGLO	57.71	1.40	40.96	0.02	nd	nd	nd	nd	nd	nd	1.29	0.01	0.04	0.03	100.00

EGO: ground by Ovahimba; GQS: Experimentally ground onto quartzite slab; EGLO: Experimentally ground (like Ovahimba), nd: not detected

XRD and ED-XRF data are in general agreement with regards the iron oxide content of the samples. Significant discrepancies between the calculated Fe_2_O_3_ and hematite and goethite concentrations concern the samples from Napier (15 and 16) and Cape Point (18 and 24), where the former is twice as high as the latter. This could be explained by a low degree of goethite and hematite crystallinity, leading to an underestimation of their concentration by the Rietveld refinement technique. XRD and ED-XRF are also concordant for K_2_O and illite/muscovite or microcline content respectively for Cape Point and Ovahimba samples.

#### SPF values

The *in vitro* SPF of the samples range from 1.9 (± 0.1) to 13.1 (± 1.1) (Table 7). Pigment powder produced by Ovahimba women consistently exhibit SPF values higher than those recorded for specimens processed experimentally by the authors. Given their high *in vitro* SPF and UVAPF values, samples 1, 5, 7, 10, 16 and 18 were subjected to *in vivo* SPF analysis. The *in vivo* SPF of the samples, applied to human skin after being mixed with clarified butter, range from 3.5 to 10.7 (Table 8). As indicated by the results obtained for subjects A, B and C, actual and average SPF values are not markedly divergent and experimental samples do display a degree of consistency in terms of their respective SPF values. Sample 18 (Cape Point—hard shale-derived red ochre) provides the highest SPF values, followed by sample 16 (Napier—medium hard shale-derived red ochre) and sample 10 (Opuwo—fine-grained red ochre).

**Table 7:** *In vivo* SPF and *in vitro* UVAPF values and critical wavelengths (CW) of ethnographic and experimental ochre powder samples.

Sample	Source	Processing method	Colour	SPF	SD	UVAPF	SD	CW	SD
1	Okamanga	EGO	Dark red	13.1	1.1	4.0	0.3	389.2	0.1
2	Okamanga	EGO	Dark red	8.6	0.6	8.0	2.7	389.1	0.1
3	Okamanga	EGO	Dark red	6.8	0.5	3.5	0.4	389.6	0.0
4	Okamanga	EGO	Dark red	7.0	0.9	3.4	0.4	389.6	0.3
5	Ovinjange	EGO	Dark red	12.4	2.6	13.7	2.8	389.4	0.0
6	Otjongoro	EGO	Dark red	6.3	1.3	6.3	1.3	389.5	0.1
7	Opuwo	GQS	Dark red	10.5	3.9	11.5	3.7	389.4	0.2
8	Opuwo	GQS	Dark red	7.3	3.2	8.8	3.5	389.7	0.1
9	Opuwo	GQS	Dark red	5.5	0.9	6.3	0.9	389.8	0.2
10	Opuwo	GQS	Dark red	8.3	1.0	10.3	1.0	389.8	0.2
11	Opuwo	GQS	Dark red	4.0	0.5	4.7	0.4	390.0	0.0
12	Opuwo	GQS	Dark red	3.8	0.3	3.3	0.3	388.2	0.3
13	Napier	GQS	Yellow	6.4	2.2	5.9	1.9	387.7	0.3
14	Blombos	GQS	Light grey	2.4	0.3	2.1	0.2	387.5	0.1
15	Napier	GQS	Light red	4.1	0.3	4.0	0.3	389.2	0.1
16	Napier	GQS	Maroon	7.7	3.1	8.0	2.7	389.1	0.1
17	De Hoop	GQS	Dark red	3.0	0.5	2.9	0.4	389.1	0.0
18	Cape Point	GQS	Orange-red	7.5	0.4	7.9	0.5	389.4	0.0
19	Napier	EGLO	Yellow	5.6	0.2	5.4	0.2	388.4	0.1
20	Blombos	EGLO	Light grey	1.9	0.1	1.8	0.1	388.4	0.2
21	Napier	EGLO	Light red	3.3	0.4	3.5	0.4	389.6	0.0
22	Napier	EGLO	Maroon	3.2	0.4	3.4	0.4	389.6	0.3
23	De Hoop	EGLO	Dark red	2.6	0.1	2.7	0.1	389.5	0.2
24	Cape Point	EGLO	Orange-red	4.6	0.3	5.2	0.3	389.7	0.1

EGO: ground by Ovahimba; GQS: Experimentally ground onto quartzite slab; EGLO: Experimentally ground (like Ovahimba)

**Table 8:** SPF ratings of ochre samples 1, 5, 7, 10, 16 and 18, mixed with clarified butter, and obtained by *in vivo* assessment on three test subjects (A, B and C).

Subject	Skin type	Sample	Source	Colour	Processing	SPF
		1	Okamanga	Dark red	Ovahimba	7.9
	II	5	Ovinjange	Dark red	Ovahimba	5.1
**A**	6–10	7	Opuwo	Dark red	Experimental	4.5
	White	10	Opuwo	Dark red	Experimental	8.8
		16	Napier	Maroon	Experimental	7.8
		18	Cape Point	Orange-red	Experimental	10.0
		1	Okamanga	Dark red	Ovahimba	7.0
	II	5	Ovinjange	Dark red	Ovahimba	5.3
**B**	6–10	7	Opuwo	Dark red	Experimental	4.5
	White	10	Opuwo	Dark red	Experimental	8.4
		16	Napier	Maroon	Experimental	8.8
		18	Cape Point	Orange-red	Experimental	10.4
		1	Okamanga	Dark red	Ovahimba	7.7
	II	5	Ovinjange	Dark red	Ovahimba	5.7
	6–10	7	Opuwo	Dark red	Experimental	3.5
**C**	White	10	Opuwo	Dark red	Experimental	9.6
		16	Napier	Maroon	Experimental	9.6
		18	Cape Point	Orange-red	Experimental	10.7

SPF values obtained by *in vivo* assessment for red ochre samples mixed with both clarified butter and with animal fat on test subject A range from 4.5 to 10.0. When mixed with animal fat, SPF values range from 5.4 to 9.8. The type of binder used, whether it is dairy derived clarified butter or subcutaneous animal fat, appears to have no marked effects on the SPF capacity of ochre. A comparison of average SPF values obtained for samples applied in dry form and with binders (clarified butter and animal fat), indicates that dry ochre powder provides a degree of protection against UVR similar to ochre powder mixed with clarified butter and with animal fat. This suggests that it is not necessary to mix ochre powder with a binder to achieve satisfactory SPF values.

### Correlations between SPF and chemical and structural characteristics

A positive correlation is observed between *in vitro* SPF values and the proportion of particles <2μm, hematite and goethite content and Fe_2_O_3_ and V_2_O_3_ normalised percentages (p = <0.05) (Table 9). A negative correlation is observed between SPF values and the granulometric data D10 to D50, clay mineral content and SiO_2_. Similar trends are observed for the *in vitro* UVAPF values.

**Table 9:** Pearson’s correlation table indicating the relationship between SPF values on the one hand and the granulometric, mineralogical and elemental composition of the pigment samples on the other hand. Data in bold emphasize prominent positive or negative correlations.

Methods	Variables	SPF	UVAPF
**Grain size**	D10	**-0.71**	**-0.60**
	D25	**-0.74**	**-0.66**
	D50	**-0.51**	**-0.57**
	D75	-0.30	-0.40
	D90	-0.29	-0.41
	<2μm	**0.72**	**0.76**
**XRD**	Goethite	**0.74**	**0.68**
	Hematite	**0.74**	**0.63**
	Quartz	**-0.51**	**-0.41**
	Illite/Muscovite	-0.39	-0.39
	Kaolinite	-0.22	-0.12
	Goethite/Hematite	**0.76**	**0.66**
	Quartz/Feldspar	**-0.52**	**-0.45**
	Clays	**-0.47**	**-0.42**
**XRF**	SiO_2_ (%)	**-0.72**	**-0.62**
	Fe_2_O_3_ (%)	**0.75**	**0.66**
	MnO (%)	-0.25	-0.13
	K_2_O (%)	-0.30	-0.18
	CaO (%)	**-0.62**	**-0.47**
	TiO_2_ (%)	-0.07	-0.09
	V_2_O_3_ (%)	**0.63**	**0.57**

**Fig 5:**
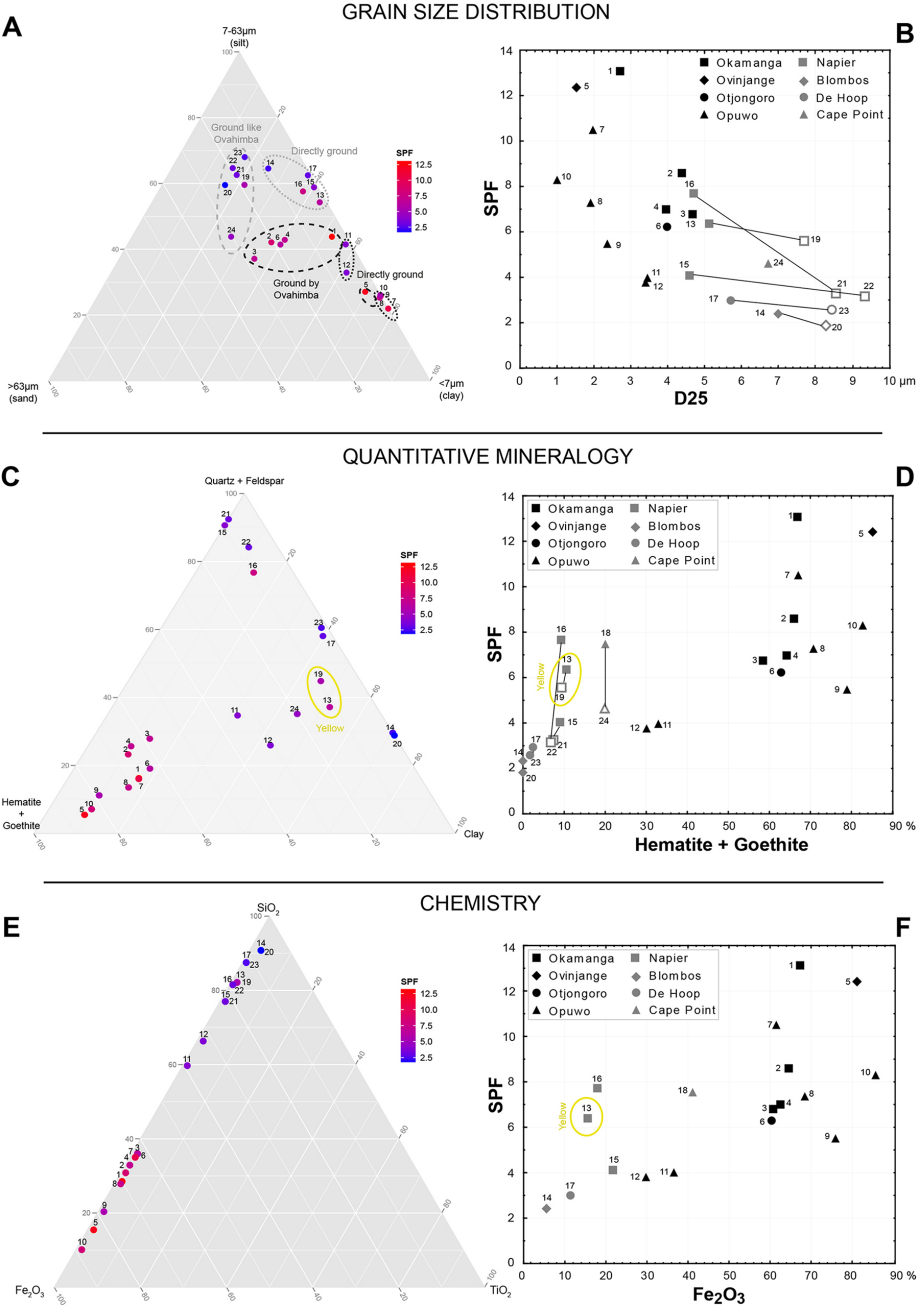
Ternary diagrams and biplots illustrating the relationship between SPF and grain size composition (A, B), mineral content (C, D) and elemental composition (E, F).

**Fig 6:**
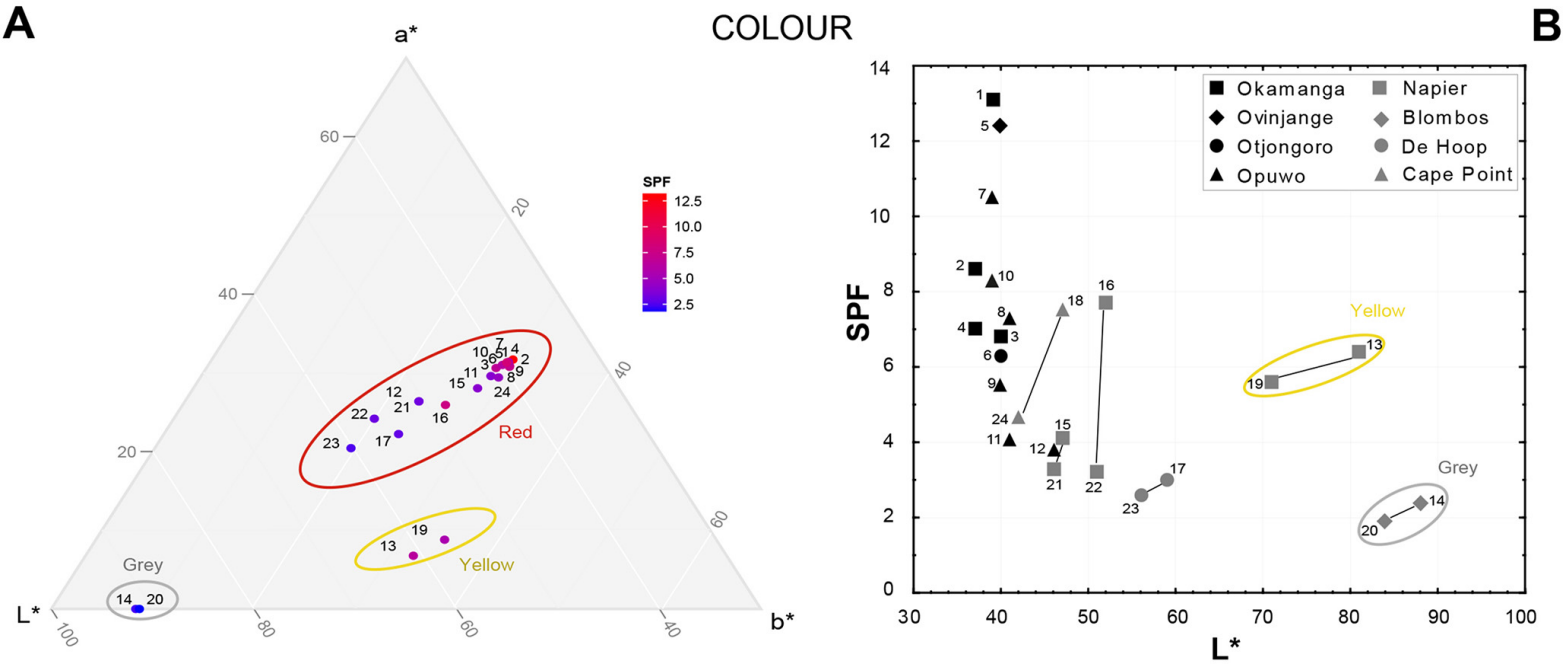
Ternary diagram (A) and biplot (B) illustrating the relationship between SPF values and ochre colour.

Figs 5 and 6 explore the relationship between SPF and grain size composition (Fig 5A and 5B), mineral content (Fig 5C and 5D), elemental composition (Fig 5E and 5F) and colour (Fig 6A and 6B). The ternary diagram correlating grain size and SPF (Fig 5A) reveals that the finer the ochre powder, the higher the photoprotective effect (SPF values). This effect becomes particularly intense when the mean size of the particles, at the first 25 centiles (D25) of the pigment grain size distribution, ranges between 1μm and 5μm (Fig 5B). SPF values derived from samples that were processed by way of different techniques are also of interest. SPF values tend to be higher when the grinding process resulted in the incidence of smaller particle sizes. Results also indicate that, when abraded directly on coarse grindstone surfaces, less homogeneous specimens, such as those from the Bokkeveld Group, can generate SPF values comparable to those obtained from homogeneous specimens, such as the Kunene Region (Ombumbuu) samples.

The ternary diagram and biplot (Fig 5C and 5D) illustrate the relationship between SPF and mineralogical composition and confirm the positive correlation between iron-rich minerals and higher SPF values. This is further supported by the results obtained for samples 11 and 12. Despite deriving from the Kunene Region, both exhibit low SPF values. This is largely due to their low hematite and goethite content. These diagrams furthermore show that samples in which iron-rich minerals are represented solely by goethite possess comparable SPF values to those in which hematite or hematite and goethite are present in the same quantities. Elemental analyses reveal a similar pattern. The correlation between the bulk normalised Fe_2_O_3_ content and *in vitro* SPF is shown to be significant, but not linear (Fig 5E and 5F). SPF is positively correlated with ochre samples in which Fe_2_O_3_ contents range between 5% and 50%, but is not further influenced once this element constitutes >60% of the sample. Conversely, TiO_2_ does not appear to influence the *in vitro* SPF values, although this element is known for its photoprotective capacity [142, 144]. This may be due to its low concentration within the analysed samples (<2%). Finally, the colorimetric properties of the samples are variably correlated with SPF values (Fig 6A and 6B). SPF is negatively correlated with lightness (L*) and positively correlated with increasing a* (red) and b* values. Samples with the brightest red hues, reflecting substantial hematite content, consistently display the highest SPF values. Goethite-rich yellow samples, and samples that have been ground directly onto coarse stone surfaces, appear to provide increased protection from UVR. This also seems to correlate with intensified a* and b* values and greater brightness, which results from this processing technique.

Iron oxide concentration and particle sizes elucidate most, but not all, of the variations observed in SPF and UVAPF values obtained by *in vitro* experiments. In many instances, other minerals appear to also influence the photoprotective efficacy of powdered ochre. A case in point involves the sample from Napier (13 and 19) which present higher SPF values than samples with similar particle size distribution (15 and 16 and 21 and 22). They differ from the latter examples in terms of higher clay mineral content, lower quartz concentration and the predominance of goethite as opposed to hematite. This suggests that variations in mineral composition do influence the overall photoprotective capacity of ochre. Sample 16 presents a similar particle size distribution and fall within the same range of Fe_2_O_3_ content than most Bokkeveld ochre samples processed experimentally using the direct method (sample 15). Nevertheless, this sample exhibits a significantly higher SPF value, which could be explained in terms of the higher concentration of kaolinite present. Whereas directly ground Ovahimba samples (7 to 10) exhibit similar particle size distribution (Fig 5A) and minor variations in mineral composition, they exhibit a surprisingly broad range of SPF values. Other variables, such as iron oxide/oxy-hydroxide crystal size and shape may also play a role in SPF value. It has been shown that crystal shape influences the optical properties of Fe oxides [150], and it is therefore predictable that it also affects the photoprotective properties of Fe-rich ochres.

## Discussion and Conclusion

Our experiments demonstrate that red ochre holds significant photoprotective capacities under both *in vitro* and *in vivo* conditions. High iron oxide content and smaller grain sizes correlate with greater photoprotective capacity (SPF). Results also confirm that it is not essential to combine powdered ochre with an organic binder to achieve optimal UVA and UVB protection. The use of binders may nevertheless be advantageous in terms of fixing ochre particles to the skin surface and therefore extending the duration of protection. The high photoprotective effect of the single yellow ochre sample was unexpected. Samples from the same location (Napier) present variable photoprotective (SPF) values which does not fit the general pattern we observe for the other samples [100, 142, 144]. Because this represents a minor proportion of the analysed instances, our discussion will focus on the broader results which suggest that the finer the ochre powder and the higher its hematite content the higher the photoprotective effect.

Although the influence of regional availability on ochre procurement strategies has been highlighted by Watts (2002) [114] and Dayet et al. (2013) [123], it is clear that MSA and LSA people deliberately selected ochre depending on its properties, and not only on its ease of acquisition. Colourimetric properties appear to have also been significant in terms of LSA and MSA ochre selection strategies. Several MSA specimens exhibit what may represent traces of streak-testing [133, 151], suggesting that ochre was consciously selected according to specific criteria. At Klasies River in South Africa, dark red ochre with unique colourimetric and mineralogical properties and therefore different from samples that were ground to extract powder, was selected for engraving purposes at 100 ka [135]. At 90 ka at Qafzeh Cave in Israel, local ochre sources were ignored in favour of more remote, but finer-grained, red sedimentary shales some 60 km from the site [152]. At 65 ka at Diepkloof Rock Shelter, and even though red ochre occurs inside the shelter, ochre from sources more than 20 km away from the site were preferentially processed [123].

Because the colour of ochre is generally indicative of elemental content and mineral composition [150], colour may have been a key criterion for ochre selection, depending on the intended purpose or applications in mind. At 100 ka, the Mousterian and Middle Palaeolithic inhabitants of the Es-Skhul (Israel) collected yellow goethite-rich ochre from 80 km away. These specimens were subsequently heated to induce colour transformations to red [153]. Based on our experimental results, one should, in addition to yellow examples, therefore expect to find substantial amounts of dark red shale-derived ochres in southern African MSA contexts. Ochre with a strong red hue does in fact appear to predominate in MSA contexts from 130 ka [114, 115, 133]. But colour-based selection does not necessarily imply a preference based on aesthetic or symbolic considerations. In addition to colour, ochre may have been selected for other physical and chemical characteristics such as grain size, processability and surface covering capacity. Cultural reasons, such as symbolic connotations to particular geological resources, may have also played a role. The widespread preference for and use of Ombumbuu red ochre, amongst the Ovahimba, suggest that colour-independent properties may also play an important role in the selection and preference of red ochre from this source.

Although ochre is attested in southern and eastern African and Levantian sites dated to at least 300 ka [151–158], its use only becomes ubiquitous during the last interglacial. During the early part of MIS 5, from 127 ka to 116 ka when temperature, rainfall and vegetation patterns are likely to have resembled those of today, human populations became archaeologically increasingly visible. The essentially only detectable behavioral signature of this transformation, the widespread exploitation of ochre, designates an increasingly complex degree of cultural complexity emerging in southern Africa at the end of MIS 5. We are cautious to argue in favour of a direct linear relationship between ochre frequency and geomagnetic excursion events and acknowledge that further research is required to substantiate this likelihood. The timing of the Blake and post-Blake geomagnetic excursions, estimated to have spanned the period between 138 ka to 100 ka [28, 29], and, more specifically, to have occurred at 120 ka to 115 ka and 100 ka to 95 ka, respectively [159], appears to both precede and coincide with the increasing prevalence of red ochre exploitation at Blombos Cave and at Klasies River Cave 1 after 120 ka [117, 133, 135]. Increased cosmogenic nuclide deposition rates, indicated by long lasting relative paleointensity records associated with full geomagnetic polarity reversals, are confirmed for both the Blake and the post-Blake events [159]. There is currently limited evidence for increasing or declining ochre exploitation following the Laschamp excursion after 40 ka. This is because we have very little information concerning the amounts and types of ochre exploited in southern Africa between 40 ka and 20 ka [160]. It is therefore difficult to assess the relationship, if any, between the suite of adaptations recorded before 60 ka and those that emerged with the LSA. Although a linear relationship between ochre frequency and insolation cannot be demonstrated at this point, the initial large-scale exploitation of red ochre does seem to coincide with the Blake excursion event after 120 ka. However, and in order to determine the exact time period in which the use of ochre was uniquely due to a cultural adaptation against insolation, one should probably consider much earlier periods, perhaps from 300 ka or more. The precise motivation for this increasing focus on red ochre is unclear. It might represent both an expansion and an exaptation of various former functions fulfilled by this material. Significantly, recent evidence derived from Sibudu Cave [161] indicate that a mixture of red ochre and casein from milk, possibly obtained from hunted lactating wild bovids, was produced by the inhabitants at 49 ka. This liquid mixture, consisting of powdered red ochre and milk, was in all probability used as a paint-like medium that could have been applied to human skin.

Our results provide a viable foundation for the emergence of ochre use, suggesting that the topical application of red ochre may have been a key innovation that functioned primarily to restrict the adverse effects of UVR exposure [100]. The skin colour of southern African MSA *Homo sapiens* is likely to have resembled the olive-brown ‘Capoid’ skin type IV (Von Luschan 16 to 21) typical of some indigenous southern African groups [76, 139]. This skin type is characteristic of more than 90% of southern San individuals, has a natural SPF of about 3.5 that of Phototype II Caucasian skin, a high tanning capacity and lower susceptibility to UVR-induced damage. In terms of avoiding vitamin D deficiencies and negating the detrimental effects of UVR, and given the correlation between adequate UVR exposure and human health, an olive-brown skin tone represents the optimal skin type for inhabitants of southern and eastern sub-Saharan Africa. It therefore stands to reason that, given the natural SPF of 3.5 for skin type IV, ochres exhibiting SPF and UVAPF values approximating or exceeding 10, represented here by the dark red ochre specimens 10, 16 and 18, are likely to have provided sufficient protection from UVR without initiating vitamin D deficiencies [139]. The topical application of red ochre may consequently have facilitated the colonisation of areas unfavourable to the constitutive skin colour of foreign immigrants, thereby increasing the ability of newcomers to compete with better adapted local populations. Subsequently, such functional innovations may have reinforced existing cultural mechanisms related to individual, intra- and inter-group identification, which in turn resulted in the emergence of ever more complex and diversified symbolic material cultures. At an individual level, ochre could have fulfilled a central role in processes of information exchange by visually enhancing the bodies, faces, hair and ornaments and attire of individuals. At the group level, ochre may have served to stimulate the formation of social and economic relationships, reinforce group solidarity and ensure cooperation or discern different statuses, achievements or affiliations amongst group members. Few Darwinian models have tried to explain the emergence of ochre use in human history. The Female Cosmetic Coalitions (FCC) model [162, 163] proposes an interesting explanatory framework for the increased exploitation of red ochre based on evolutionary constrains and the symbolic embodiment of relationships between men and women. The sunscreen model, proposed here, is not in contradiction with the FCC model in the sense that it may well explain the emergence of the phenomenon at a stage when the various symbolic functions of ochre were not yet in place. While it is difficult to establish a precise scenario for the emergence of such an innovation, one could envision a situation in which the habitual use of red ochre as a sunscreen may have originally arisen locally, perhaps in response to changing UVR exposure rates produced by orbital climatic cycles, and subsequently provided an adaptive advantage for migrating populations. It is likely that the use of ochre as a sunscreen emerged repeatedly and that this innovation periodically vanished and reoccurred, a hypothesis consistent with the chronological and geographic discontinuity in ochre occurrences during this time span. We do not know, at present, whether such applications may have facilitated the latitudinal displacement of populations and whether it coincided with symbolic connotations. It can, however, be argued that the increase in ochre use corresponding to the beginning of the last interglacial in all probability reflects a gradual, albeit significant, change of magnitude in the role of ochre in human culture, which has not come to an end since it emerged [139].

Combined with experimental replication and multi-proxy analyses, ethnographic examples of red ochre exploitation enabled the identification of noteworthy ancestral functions, while also revealing the extraordinary degree of social complexity that it has contributed to since its initial exploitation. Although the transition in archaeology from data to social interpretation is challenging, the importance of ochre exploitation during the MSA can only be expressed in terms of complex multi-functional explanations. This is so because the advantages offered by the habitual exploitation of ochre during the evolution of *Homo sapiens* were numerous. Although exclusively functional uses of ochre are rare, it can be argued that they may have been more common in the past and that the use of ochre for symbolic purposes in ethnographic contexts does not imply similar use by early MSA humans. Ochre could also have functioned symbolically before the emergence of anatomically modern *Homo sapiens* after 195 ka [164]), but it may in fact have had several functional uses early on which subsequently evolved to comprise both symbolic significance and functional value. The hypothesized and confirmed functional applications of red ochre should not therefore be viewed as in contradiction with a symbolic use of ochre, but instead as indicative of the possible exploitation and extensive application of a raw material with remarkable colourimetric, structural and chemical properties during the MSA.
